# Intravenous leiomyomatosis manifesting as a cardiac mass: a case report

**DOI:** 10.3389/fcvm.2026.1791911

**Published:** 2026-06-02

**Authors:** Haoxuan Deng, Qiyue Zhu, Wei Qiu, Yunyan Zhang, Junyi Hua

**Affiliations:** Second Affiliated Hospital, Zhejiang Chinese Medical University, Hangzhou, China

**Keywords:** cardiac mass, differential diagnosis, intravenous leiomyomatosis, multidisciplinary diagnosis, multidisciplinary treatment

## Abstract

This article reports a 45-year-old woman with intravenous leiomyomatosis (IVL) who presented with chest tightness and was initially diagnosed as a cardiac mass. Multimodal imaging, including contrast-enhanced pelvic CT and transthoracic echocardiography, revealed IVL extending from the pelvic veins through the inferior vena cava into the right atrium. The patient underwent multidisciplinary two-stage surgical resection. Histopathological and immunohistochemical examinations confirmed IVL, with positive *α*-SMA, desmin, CD10, ER, PR and negative HMB45, Melan-A, and S100. Only short-term postoperative imaging data are available at the time of writing; long-term follow-up is ongoing, and the lack of extended follow-up data represents a limitation given the known recurrence risk of IVL. This case emphasizes the rarity of IVL presenting as a cardiac mass and the value of multidisciplinary diagnosis and treatment.

## 1 Introduction

Intravenous Leiomyomatosis (IVL) is a rare benign smooth muscle tumor with a low overall incidence (1). A retrospective analysis from Peking Union Medical College Hospital reported that among 30,757 patients treated for uterine fibroids, only 76 cases (0.25%) were diagnosed with IVL (2). IVL predominantly affects middle-aged women, with approximately 80.4% of patients between 36 and 55 years of age (3, 4). IVL most commonly in the uterus or urinary tract but also been reported in uncommon locations, including the thoracic cavity, breast, cavernous sinus, oral cavity, and perianal region, where it may cause local complications due to compression of adjacent nerves and vessels (5–8). Presentation with primary cardiac symptoms is particularly rare. In such cases, patients often lack early pelvic manifestations, substantially increasing the risk of misdiagnosis.

Cardiac involvement in IVL typically arises from smooth muscle cells of the uterus or venous walls, with tumors extending intravascularly along the venous system to the heart (9). This distinctive growth pattern results in clinical and imaging features that closely resemble those of cardiac myxomas or thrombi. Cardiac myxomas, the most common primary cardiac tumors, often occur in the left atrium (10) and display characteristic imaging fundings, such as well-circumscribed, homogeneous masses attached to the interatrial septum near the fossa ovalis. However, distinguishing IVL from myxomas or clinical presentation and imaging remains challenging (11).

Misdiagnosed of IVL as thrombotic disease may lead to inappropriate anticoagulation therapy, delaying definitive surgical intervention and allowing further tumor progression. In advanced cases, tumor extension from the inferior vena cava into the right atrium, right ventricle, or pulmonary artery may occur, resulting in life-threatening complications such as tumor rupture and pulmonary embolism (12). Therefore, this article presents a representative cases and reviews the relevant literature to improve clinical recognition of IVL presenting as an atypical cardiac masses, with the aim of reducing misdiagnoses and optimizing management strategies.

## 2 Case information

### 2.1 Medical history

The patient was a 45-year-old women admitted with a chief complaint of chest discomfort for two weeks, which was exertion-related and accompanied by cough and sputum production. Two weeks prior to admission, the patient first experienced chest discomfort after daily activities. The symptoms were relieved by rest but recurred with exertion. During this period, she reported frequent coughing with sputum production. Shortly before admission, she noticed a small amount of menstrual-like blood during urination. She denied chest pain, chills, fever, or other systemic symptoms.

Past medical history: The patient has had irregular menstruation over the preceding two months. Two days prior to admission (August 12, 2025), she observed several drops of menstrual blood during urination. She had previously undergone cholecystectomy and denied other significant medical conditions, blood transfusions, or known drug allergies. Menstrual history revealed a cycle length of 35−40 days with a duration of 5–7 days. The last menstrual period began on August 11, 2025. On physical examination at admission, vital signs were stable. Cardiac auscultation revealed a systolic murmur with a regular rhythm. Breath sounds were midly coarse bilaterally, without dry or wet rales. No lower extremity edema was observed.

### 2.2 Auxiliary examination

**Figure 1:**
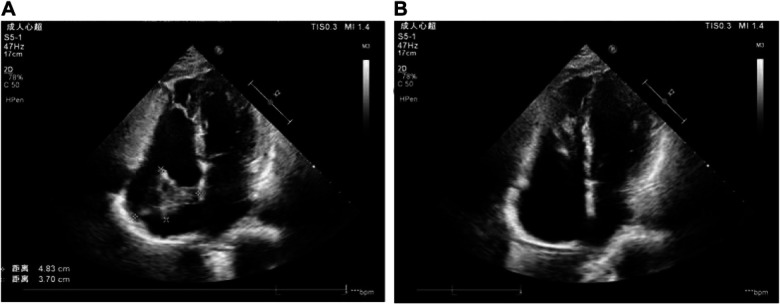
Echocardiography (August 6, 2025). **(A)** Apical four-chamber view; **(B)** Apical four-chamber view (supplemental view).

Transthoracic echocardiogram demonstrated right atrial enlargement, measuring approximately 4.2 cm in width and 6.1 cm in length. A slightly hyperechoic mass measuring approximately 4.8  ×  3.7 cm was identified within the right atrium, with an irregular shape and heterogeneous echogenicity. During diastole, the mass nearly prolapsed into the right ventricle. Atrial myxoma was considered, although other tumors could not be excluded. Mild to moderate tricuspid regurgitation was observed, with mildly elevated pulmonary artery pressure. The peak TR velocity was 3.0 m/s, corresponding to an estimated pulmonary artery systolic pressure of 41 mmHg and an LVEF of 65% (Figure 1).

**Figure 2:**
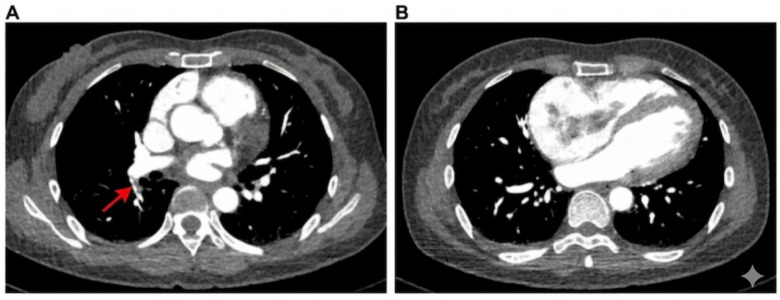
Total aortic computed tomographic angiography. **(A)** Pulmonary embolism in both lower lungs; **(B)** Right heart mass.

The CTA of the entire aorta showed a right heart mass and multiple low-density filling defects in the lower pulmonary arteries, raising concern for differentiation between an atrial tumors and thromboembolism. The right iliac vein appeared poorly visualized, suggesting possible thrombosis. Multiple enlarged and tortuous vascular structures were observed on the left side of the pelvis, raising suspicion for an arteriovenous malformation (Figure 2).

**Figure 3:**
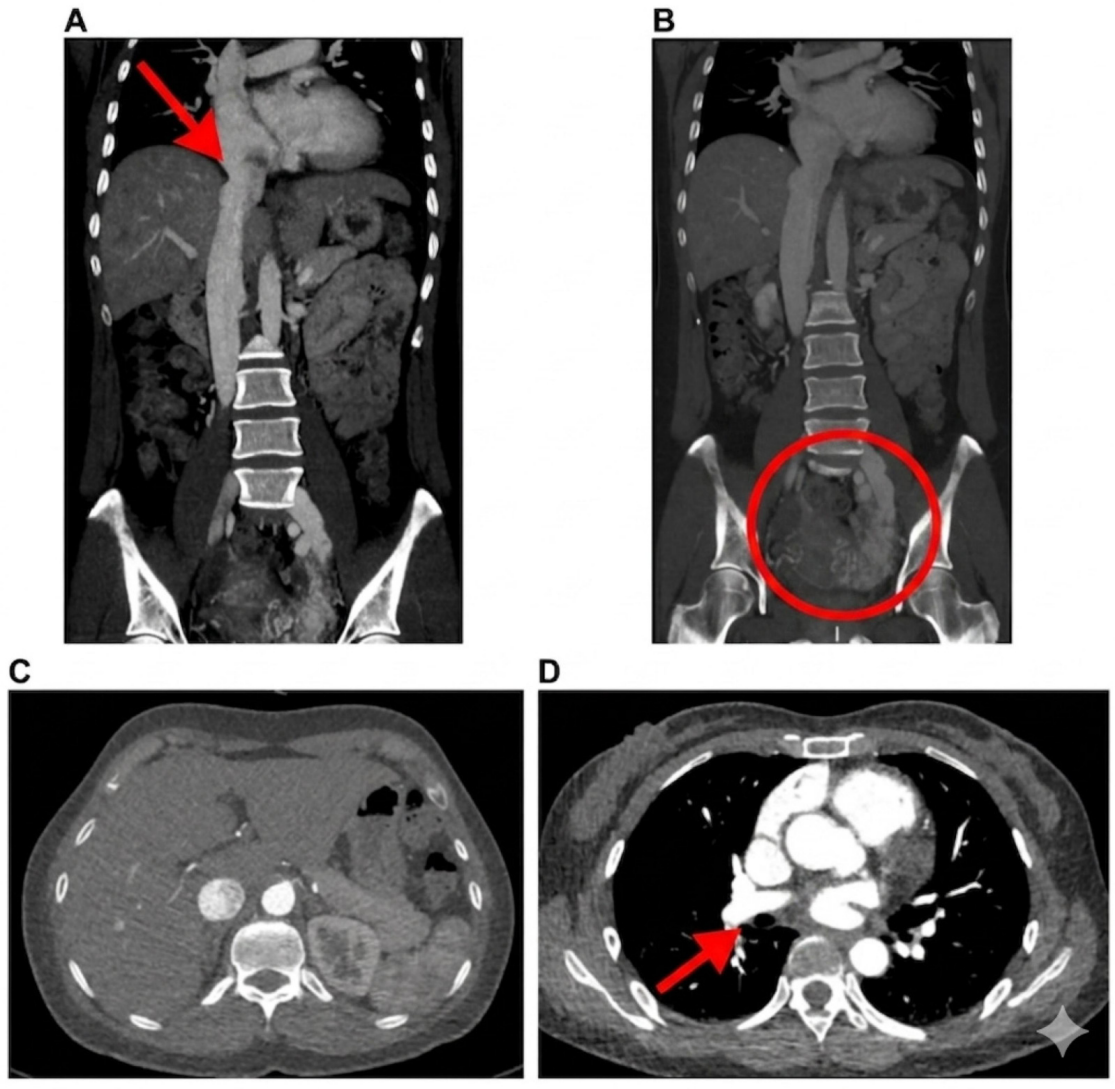
Preoperative inferior vena cava computed tomographic angiography. **(A)** Inferior vena cava CTA sagittal reconstruction section; **(B)** Inferior vena cava CTA sagittal reconstruction section (another perspective); **(C)** Inferior vena cava CTA pelvic axial (cross section) section; **(D)** Inferior vena cava CTA axial section of the chest.

**Figure 4:**
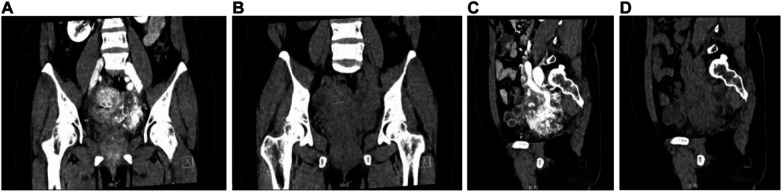
Preoperative pelvic enhanced computed tomography. **(A)** Pelvic coronal reconstruction section (uterus overall morphology + fibroids); **(B)** Pelvic coronal reconstruction section (right adnexal area); **(C)** Pelvic contrast-enhancement coronal section (vascular lesions on the left side of the pelvis); **(D)** Pelvic contrast-enhanced coronal section (leiomyoma enhancement manifestation).

CTA of the inferior vena cava demonstrated abnormally dilated, rortuous, and clustered vessels on the left side of the pelvis, further suggesting vascular malformation (Figure 3). Pelvic contrast-enhanced CT showed an irregularly shaped uterus with multiple fibroids, the largest measuring approximately 3 cm in diameter, with clear margins and mild enhancement. An intrauterine device was present. A low-density lesion measuring approximately 22 × 28 mm with clear boundaries was identified in the right adnexal region, consistent with a cyst. Vascular-like enhancing lesions with focal dilation were noted in the left pelvis, along with suspicious filling defects in the left iliac vein and inferior vena cava, raising concern for a smooth muscle tumor with intravascular involvement. Pelvic ultrasonography comfirmed uterine fibroids recommended further transvaginal evaluation. Right ovarian cystic enlargement was classified as O-RADS category 1 (Figure 4). Abdominal ultrasonography revealed hepatic steatosis and postoperative changes consistent with prior cholecystectomy, without biliary dilation. The pancreas and spleen were unremarkable.

### 2.3 Diagnosis and treatment process

Based on the clinical presentation and imaging findings, the preliminary diagnosis includes: ① intracardiac mass (right atrium, right ventricle); ② left pelvic smooth muscle tumor with vascular involvement; ③ pulmonary embolism; ④ mass in the right iliac vein and inferior vena cava.

A multidisciplinary team formulated a two-stage surgical strategy, consisting of initial management of the pelvic lesions followed by resection of the cardiac and intravascular tumors. After obtaining informed consent, the first-stage surgery was performed on August 18, 2025. Under general anesthesia, laparoscopic total hysterectomy, bilateral salpingo-oophorectomy, and pelvic adhesion lysis were completed. Intraoperative and perioperative cardiac function was closely monitored. Diuretics and vasoactive agents were administered to maintain hemodynamic stability, and anticoagulation was carefully adjusted to reduce the risk of embolization. Blood products were prepared, and postoperative intensive care monitoring was arranged.

**Figure 5:**
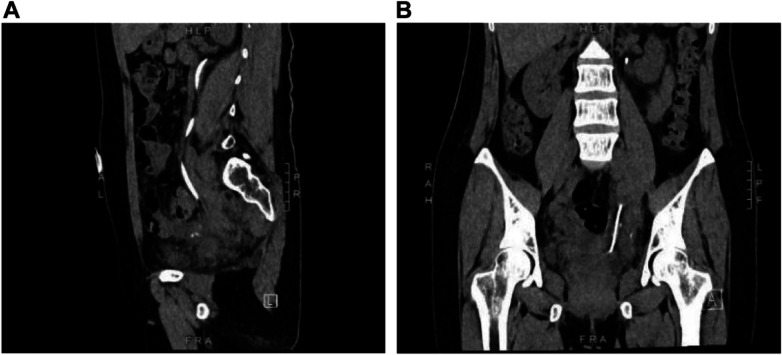
Postoperative abdominal non-contrast computed tomography (August 27, 2025). **(A)** Abdominal CT sagittal reconstruction section; **(B)** Abdominal CT coronal reconstruction slice.

**Figure 6:**
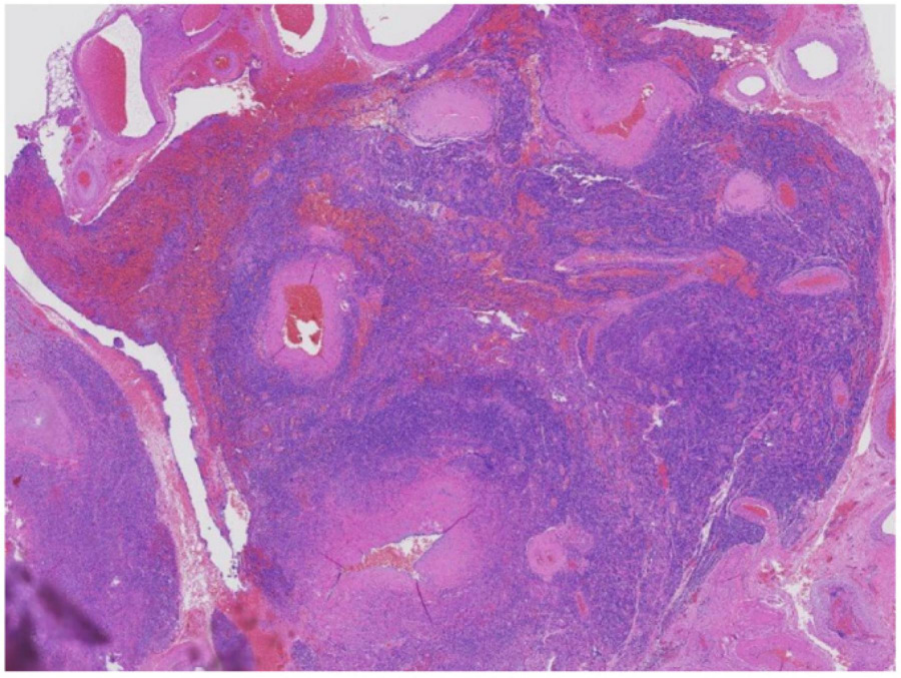
Pathological results of primary surgery (August 29, 2025).

Intraoperatively, multiple uterine fibroids were identified. The left pelvic region exhibited thickened vessels and multiple nodular tumors adherent to surrounding tissues. All visible lesions were meticulously dissected and completely excised, with specimens sent for frozen-section analysis. Postoperative abdominal CT revealed postoperative changes with localized vascular tortuosity and exudative findings in the surgical field and pelvis (Figure 5). Final pathological examination demonstrated uterine smooth muscle tumor with areas of increased cellularity and vascular proliferation. Additional findings included endometrium, chronic cervicitis with Nabothian cyst formation, an ovarian inclusion cyst, and focal stroma vascular proliferation in the contralateral ovary. The left pelvic mass was confirmed as a smooth muscle tumor with similar histological features. Immunohistochemical results showed: *α*-SMA (+), Desmin (+), CD10 (+), Ki-67 (1% +), Caldesmon (partially +), ER (+), PR (+), CD34 (vascular +), STAT6 (-), HMB45 (-), Melan-A (-), TFE3 (-), CK-PAN (scattered +), S100 (-), SOX10 (-), PAX8 (-) (Figure 6).

**Figure 7:**
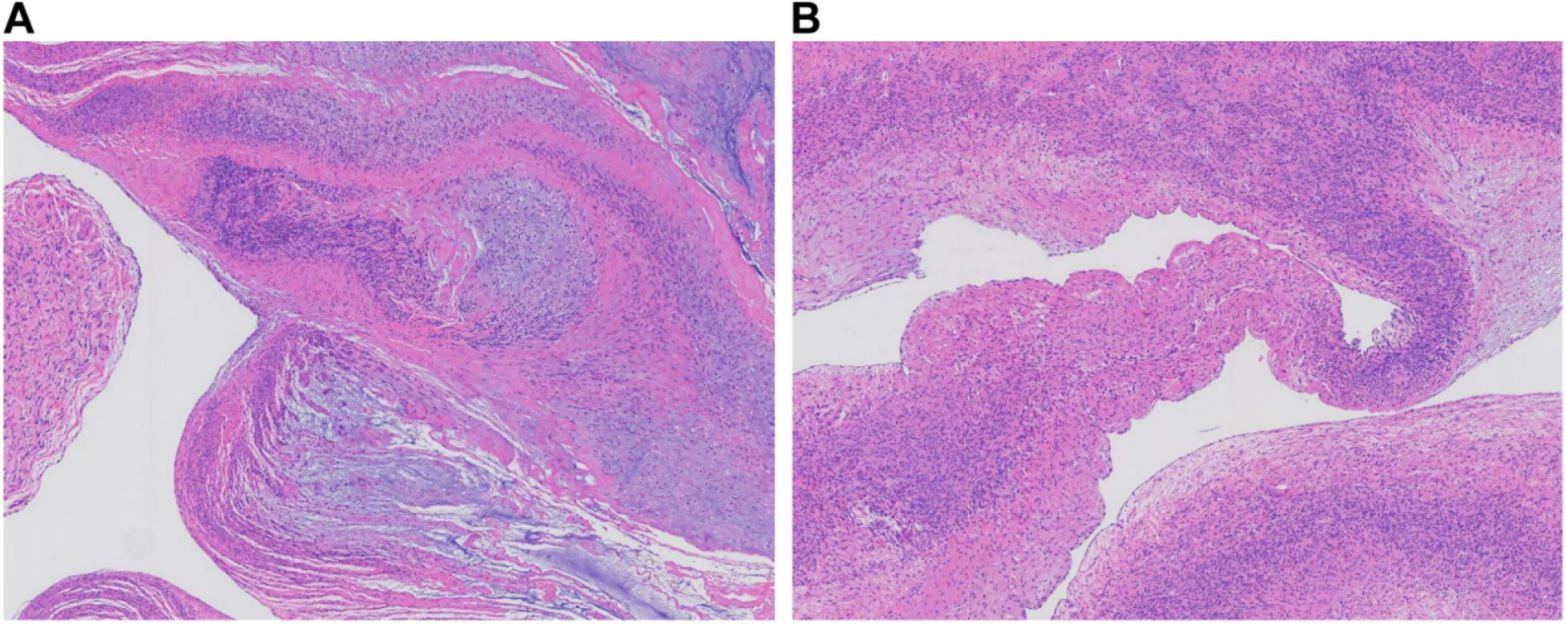
Postoperative pathological results of second-stage surgery (September 4, 2025). **(A)** HE staining sections (mucous degeneration, hyaline change areas); **(B)** HE staining section (irregular dilation and hyperplasia area of blood vessels).

One week later, with stable clinical status, the second-stage surgery was performed on August 26, 2025. Under cardiopulmonary bypass, the intravascular smooth muscle tumor extending from the inferior vena cava into the right atrium was completely resected via a median sternotomy. After aortic cross-clamping and induction of crystalloid cardioplegic arrest, the right atrium was opened to expose the tumor, which was found to be continuous with the intravascular lesion originating from the inferior vena cava. The tumor was carefully dissected from the endocardial surface and caval wall, with complete removal of the entire intracardiac and intravascular tumor segment. Careful inspection confirmed no residual tumor, and the right atrial wall and inferior vena cava were repaired with continuous sutures to restore vascular integrity and hemostasis. We did not employ deep hypothermic circulatory arrest in this procedure. Postoperatively, the patient was weaned uneventfully from cardiopulmonary bypass and transferred to the cardiac intensive care unit for monitoring. Pathological examination confirmed an intravascular smooth muscle tumor with myxoid degeneration, hyalinization, and irregular vascular proliferation. Immunohistochemical results were: *α*-SMA (+), Desmin (+), Caldesmon (+), CD31 (vascular +), CD34 (vascular +) (Figure 7).

### 2.4 Follow-up

**Figure 8:**
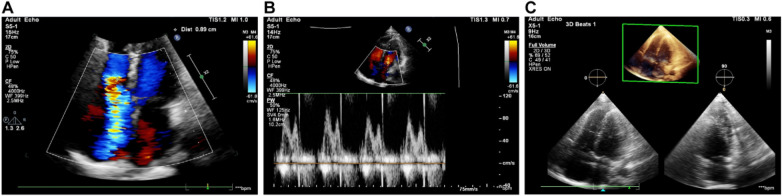
Postoperative follow-up echocardiographic results (August 31, 2025). **(A)** Color Doppler imaging of the apical four-chamber view; **(B)** The spectrum Doppler image of tricuspid regurgitation; **(C)** Two-dimensional ultrasound + three-dimensional ultrasound section.

Postoperative echocardiography showed mild right atrial enlargement (approximately 4.05 cm  × 5.66 cm) with preserved right heart function. Moderate tricuspid regurgitation persisted, with regurgitant jet width of approximately 0.89 cm and a peak velocity of 2.93 m/s, corresponding to an estimated pulmonary artery pressure of 37 mmHg. Mild mitral regurgitation was present, and LVEF improved to 58.7% (Figure 8). After discharge, a long-term follow-up plan was established, including regular echocardiograms, pelvic ultrasonography, and CT imaging to monitor for recurrence. Periodic hormonal evaluations were also planned to assess endocrine status and guide subsequent treatment.

## 3 Discussion

This case describes a middle-aged woman who initially presented with a cardiac mass and was ultimately diagnosed with intravascular leiomyomatosis (IVL) based on comprehensive imaging and pathological evaluation. Although the patient mainly complained of exertional chest discomfort, echocardiography revealed a mass in the right atrium. Following the structured framework for cardiac mass differential diagnosis proposed by Bartoli (13), cardiac masses are divided into primary cardiac tumors, secondary cardiac involvement, and non-neoplastic lesions, and this classification was applied sequentially to evaluate the right atrial mass in the present patient. Right atrial myxoma, a common primary cardiac tumor, was excluded because the mass was irregular, heterogeneous, non-pedunculated, and had no attachment to the interatrial septum, which was inconsistent with typical myxoma features (14). Intracardiac thrombus was ruled out due to the absence of atrial fibrillation, valvular disease, or other thrombotic risk factors, as well as the demonstration of a continuous intraluminal lesion extending from the pelvic veins to the right atrium on imaging (15). Cardiac metastasis was also excluded as the patient had no history of malignant disease, systemic cancer-related symptoms, or radiological signs of malignancy, and subsequent pathology confirmed a benign neoplasm (16). The mass was therefore determined to be neither a primary cardiac tumor nor a metastatic or non-neoplastic lesion, but benign secondary cardiac involvement resulting from intravascular extension of IVL. Of particular importance, pelvic contrast-enhanced CT served as the pivotal diagnostic examination that redirected the entire workup, revealing uterine fibroids, abnormal pelvic vascular lesions, and continuous filling defects extending from the iliac vein through the inferior vena cava into the right atrium; this continuous venous pathway confirmed the diagnosis of IVL and represents a key learning point that targeted pelvic imaging is essential for middle-aged women presenting with a right-sided cardiac mass to avoid misdiagnosis.

The clinical condition was complicated by concomitant cardiac mass, pulmonary embolism, and pelvic vascular abnormalities. After multidisciplinary team (MDT) discussion involving gynecology, vascular surgery, cardiac surgery, radiology, and oncology, a staged surgical strategy was selected (17). The first stage surgery focused on removal of the pelvic lessions, aiming to reduce tumor burden and eliminate the primarly source while the patient's cardiac function remained stable. After adequate recovery, the second-stage procedure allowed complete resection of the tumor extending into the inferior vena cava and right atrium, avoiding the risks associated with prolonged combined surgery and excessive operative trauma.

IVL is characterized by intraluminal growth along the venous system, typically extending from uterine veins through the iliac veins and inferior vena cava to the right atrium (18). This growth pattern was clearly demonstrated in this case by pelvic imaging, inferior vena cava CTA and echocardiography. IVL predominantly affects women of reproductive age and is hormone dependent, with estrogen and progesterone receptors promoting tumor proliferation and migration (19–22). Approximately 70% of patients have a history of uterine fibroids, suggesting a close association between the two conditions (23).

When IVL presents initially as a cardiac mass, clinical manifestations are often nonspecific, including chest discomfort and dyspnea, and may be misdiagnosed as heart failure, pulmonary embolism, or primary cardiac tumors. Some patients fail to report a gynecological history at initial presentation, further delaying diagnosis (24). Echocardiography often identifies a right atrial mass, leading to misdiagnosed as myxoma (25). Unlike myxomas, IVL typically lacks a pedicle attached to the interatrial septum (26). Although CTA (27) and MRI (28) can demonstrate tumor continuity along the venous system, differentiation from thrombus or malignant tumors remains challenging. Extensive involvement of the right atrium, ventricle, or pulmonary artery increases surgical difficulty and recurrence risk (29). Previous studies have reported relatively high recurrence rates after IVL resection, with occasional distant metastasis (30). In cases combined with pelvic arteriovenous fistula, abnormal shunting further increases cardiac burden and worsens prognosis (31).

Accurate diagnosis relies on careful medical history and multimodal imaging. Echocardiography is a useful screening tool for evaluating tumor mobility, valvular involvement, and hemodynamic effects (9), but CT and MRI are often required for further characterization (32). MRI provides superior soft tissue contrast and spatial resolution, aiding assessment of tumor extent and differentiation from thrombus (33, 34). CT can clearly depict the venous extension pathway from the pelvis to the heart, as demonstrated in this case (35). Emerging techniques such as dual-energy CT (DECT) may further improve diagnostic accuracy (36).

Pathological examination remains the gold standard for diagnosis. In this case, immunohistochemical findings were highly consistent with intravenous leiomyomatosis and provided critical diagnostic clues. Positivity for *α*-SMA, desmin, and caldesmon confirmed the smooth muscle lineage of the tumor (37–39). CD10 positivity strongly supported a uterine origin, consistent with the pelvic source demonstrated on imaging (40). Positive expression of estrogen receptor (ER) and progesterone receptor (PR) verified the hormone-dependent nature of the neoplasm, providing a rational basis for postoperative hormonal therapy to reduce recurrence risk. Meanwhile, negative staining for HMB45, Melan-A, S100, SOX10, and STAT6 effectively excluded malignant melanoma, peripheral nerve sheath tumors, and solitary fibrous tumors, confirming the benign biological behavior of the lesion (41, 42). These results were concordant between the primary pelvic tumor and the second-stage intracardiac and intravascular tumors, further supporting the diagnosis of intravenous leiomyomatosis with continuous extension into the right heart. Molecular mechanisms involving cell adhesion, matrix degradation, and abnormal angiogenesis may play contribute to intravascular invasion and tumor progression (43). sICAM-1 may participate in IVL progression by regulating leukocyte migration and inflammatory responses (44). Enhanced MMP expression and activity promote extracellular matrix degradation, disrupt vascular integrity, and facilitate intravascular invasion and cardiac extension of tumor cells (45). These mechanisms are consistent with the pathological features observed in this case, including spindle-shaped cells arranged in bundles, elongated nuclei, no atypia, and absence of atypia or mitotic figures, characteristic of leiomyoma. IVL cells may also evade immune surveillance, as platelet endothelial cell adhesion molecule (PECAM) expressed by endothelial and smooth muscle cells can impair immune cell recruitment (46), while *α*-SMA–positive tumor-associated fibroblasts and abnormal tumor vasculature further limit immune infiltration and promote angiogenesis (47, 48).

The primary goal in treating cardiac masses from intravascular leiomyoma is complete tumor excision to prevent recurrence and cardiac complications. Surgical approaches include single-stage or multi-stage procedures. Single-stage surgery suits patients with well-defined tumors and good physical condition, enabling removal of all lesions at once, reducing anesthetic risk, and shortening treatment time (49, 50). Multi-stage surgery is preferred for extensive tumors or patients with limited tolerance, often involving cardiopulmonary bypass or deep hypothermic circulatory arrest to ensure complete tumor resection while minimizing complications (51, 52). Abdominal pelvic procedures typically involve total hysterectomy and adnexectomy to eliminate the primary tumor source (53). Clinical reports describe a staged approaches in which the cardiac tumor is first under excised via median sternotomy under low-temperature circulatory arrest, followed by adjuvant endocrine therapy, and subsequently, residual pelvic tumors and ovaries are removed (51). Postoperative management requires individualized anticoagulation due to enhanced hypercoagulability from vascular injury (54). Hormonal therapy with GnRH agonists or aromatase inhibitors is effective for residual or hormone receptor–positive lesions, controlling tumor growth and reducing recurrence. For instance, a patient receiving two-stage surgery combined with GnRH agonist therapy remained recurrence-free for 15 months (51), while another patient treated with letrozole experienced significant tumor reduction and remained asymptomatic (55). Therefore, postoperative hormonal therapy is recommended for patients at high risk of recurrence or when complete excision is not feasible (56). Although current short-term outcomes demonstrate favorable efficacy in controlling tumor progression and alleviating clinical symptoms, the available evidence remains insufficient to fully characterize long-term oncological behavior and late recurrence patterns. Long-term follow-up is still ongoing; only short-term data are available at present, which limits the assessment of late recurrence.

In summary, for patients with cardiac masses, especially middle-aged women, clinicians should consider pelvic and vascular evaluations, menstrual and uterine history, and imaging such as echocardiography, CTA, and MRI to identify intravascular leiomyoma and prevent misdiagnosis. Treatment requires multidisciplinary collaboration, with staged surgery reducing risk and ensuring complete tumor removal. There is no standard for hormonal adjuvant therapy, and follow-up relies mainly on imaging. Future work should focus on advanced imaging, molecular mechanisms, targeted therapies, standardized long-term follow-up, and optimized hormonal regimens to reduce recurrence and improve outcomes.

## Data Availability

The original contributions presented in the study are included in the article/Supplementary Material, further inquiries can be directed to the corresponding author.

## References

[1] Dalainas I. Vascular smooth muscle tumors: review of the literature. Int J Surg. (2008) 6(2):157–63. 10.1016/j.ijsu.2007.03.004 17531562

[2] Ge Z Wang Y Wang Y Li W Yang X Li J. Diagnostic experience of intravenous leiomyomatosis with emphasis on conventional ultrasonography imaging: a single-center study. Front Oncol. (2023) 13:1203591. 10.3389/fonc.2023.1203591 37492474 PMC10364609

[3] Chen J Bu H Zhang Z Chu R Qi G Zhao C. Clinical features and prognostic factors analysis of intravenous leiomyomatosis. Front Surg. (2023) 9:1020004. 10.3389/fsurg.2022.1020004 36793517 PMC9922872

[4] Zhao XY Feng ZY Liu KR. Clinical features and diagnosis of 51 cases of leiomyomatosis in uterine veins. Prog Obstet Gynecol. (2021) 30(9):679–82.

[5] Xu CQ Zhang J. A rare case of perianal blood vessel smooth muscle tumor. Chin J Coloproctol. (2025) 45(5):76–7.

[6] Liu MD Zheng JJ Song DL Zhang XJ. Endoscopic resection of an angioleiomyoma in the cavernous sinus: a case report and literature review. Chin J Minim Invasive Neurosurg. (2025) 29(5):305–8.

[7] Zhang JX Li Y Ning Z Guo X Wang Y Xie SL. A case of vascular smooth muscle tumor of the female breast. J Dalian Med Univ. (2025) 47(2):177–85.

[8] Qi XB Jiang HJ He Q. Nursing care for the combined tumor resection surgery involving vascular smooth muscle tumors affecting the inferior vena cava and the right heart. Nurs Rehabil J. (2022) 21(3):65–7.

[9] Han X Nie F Li J Zhao Y Wang X. Intravenous leiomyomatosis involving the right heart: comprehensive evaluation using echocardiography combined with abdominal ultrasonography. Echocardiography. (2023) 40(8):852–5. 10.1111/echo.15565 37270687

[10] Rafajlovski S Ilić R Gligić B Kanjuh V Tatic V Ristic A. Impact of heart myxoma localization upon its clinical course and outcome. Vojnosanit Pregl. (2012) 69(3):270–6. 10.2298/VSP100326002R 22624416

[11] Raicea VC Suciu H Raicea AD Macarie GC Mezei T Maier MS. Giant left atrial myxoma – literature review and case presentation. Rom J Morphol Embryol. (2021) 62(2):361–8. 10.47162/RJME.62.2.02 35024724 PMC8848268

[12] Han Y Chung YJ Shin I Park JY Shim S Hijazi A. Intravenous leiomyomatosis misdiagnosed with large thrombosis in inferior vena cava. Taiwan J Obstet Gynecol. (2021) 60(2):367–9. 10.1016/j.tjog.2021.01.019 33678345

[13] Bartoli L Angeli F Stefanizzi A Fabrizio M Paolisso P Bergamaschi L. Genetics and clinical phenotype of Erdheim-Chester disease: a case report of constrictive pericarditis and a systematic review of the literature. Front Cardiovasc Med. (2022) 9:876294. 10.3389/fcvm.2022.876294 36035941 PMC9403274

[14] Vasilyev NV Krakhmal NV Vtorushin KS Stepanov IV Vtorushin SV. Cardiac myxoma: biological features, morphology, differential diagnosis. Arkh Patol. (2024) 86(6):74–81. 10.17116/patol20248606174 39686901

[15] Cresti A García-Fernández MA Sievert H Mazzone P Baratta P Solari M. Prevalence of extra-appendage thrombosis in non-valvular atrial fibrillation and atrial flutter in patients undergoing cardioversion: a large transoesophageal echo study. EuroIntervention. (2019) 15(3):e225–30. 10.4244/EIJ-D-19-00128 30910768

[16] Swierz J Poznański J Stawarz B. Metastasis of penile cancer to the heart in a 20-year-old patient. Wiad Lek (Warsaw, Poland: 1960). (1992) 45(7-8):314–6. 1462597

[17] Ma GT Miao Q Liu XR Zhang CJ Zheng YH Shao J. Surgical treatment strategies for venous smooth muscle tumor disease involving the right heart chambers. Acta Acad Med Sinica. (2016) 38(4):438–43. 10.3881/j.issn.1000-503X.2016.04.013 27594158

[18] Zhang T Zhang XM Li QL. Intravenous leiomyomatosis with intracardiac extension: a case report. Beijing Da Xue Xue Bao. Yi Xue Ban=J Peking Univ Health Sci. (2008) 40(6):642–4. 19088839

[19] Kirschen GW AlAshqar A Miyashita-Ishiwata M Reschke L El Sabeh M Borahay MA. Vascular biology of uterine fibroids: connecting fibroids and vascular disorders. Reproduction. (2021) 162(2):R1–R18. 10.1530/REP-21-0087 34034234 PMC8320308

[20] Bulun SE Yin P Wei J Zuberi A Iizuka T Suzuki T. Uterine fibroids. Physiol Rev. (2025) 105(4):1947–88. 10.1152/physrev.00010.2024 40214304 PMC12419501

[21] Podoleanu C Balos S Stolnicu S. An unusual shaped mass filling the right cardiac chambers in a woman with a vascular malformation. Pol J Pathol. (2022) 73(3):281–2. 10.5114/pjp.2022.124495 36734443

[22] Das CJ Rathinam D Manchanda S Srivastava DN. Endovascular uterine artery interventions. Indian J Radiol Imaging. (2017) 27(4):488–95. 10.4103/ijri.IJRI_204_16 29379246 PMC5761178

[23] Wang Q Liu H Feng W. Unraveling the challenges of intravenous leiomyomatosis: a retrospective analysis of 11 cases. Arch Gynecol Obstet. (2024) 309(2):621–9. 10.1007/s00404-023-07308-x 38085353 PMC10808418

[24] Su K Zhang Y Cai YJ Zhang YY Zhao ML Guo RX. Clinical analysis of 57 cases of intravascular smooth muscle tumor. Chin J Pract Gynecol Obstet. (2024) 40(6):653–6.

[25] Fornaris RJ Rivera M Jiménez L Maldonado J. Multimodality evaluation of intravenous leiomyomatosis: a rare, benign but potentially life-threatening tumor. Am J Case Rep. (2015) 16:794. 10.12659/AJCR.894939 26546569 PMC4644017

[26] Jang KH Shin DH Lee C Jang J-K Cheong S Yoo S-Y. Left atrial mass with stalk: thrombus or myxoma? J Cardiovasc Ultrasound. (2010) 18(4):154. 10.4250/jcu.2010.18.4.154 21253367 PMC3021896

[27] Gui T Qian Q Cao D Yang J Peng P Shen K. Computerized tomography angiography in preoperative assessment of intravenous leiomyomatosis extending to inferior vena cava and heart. BMC Cancer. (2016) 16(1):73. 10.1186/s12885-016-2112-9 26858203 PMC4746779

[28] Santhosh J Al-Mughairfi S Al-Ghaithi H Al-Hilal Z Al-Maqbali RH Al-Salmi A. Clinico-radio-histopathological correlation of leiomyoma variant, STUMP, and sarcoma: a retrospective study. Oman Med J. (2025) 40(1):e715. 10.5001/omj.2025.49 40337319 PMC12056707

[29] Liu Y Yu Z Wang R Chen K-S Yue S-W Gao X-M. Diagnostic value of dual-energy CT and clinicopathological and imaging feature analysis of mixed endometrial stromal and smooth muscle tumors with intracardiac extension. Front Cardiovasc Med. (2022) 9:917399. 10.3389/fcvm.2022.917399 36187004 PMC9521406

[30] Zhang TT Zhou JY Li HL Zhang X-L Chang L. Ultrasound diagnostic value and clinical analysis of 61 uterine intravenous leiomyomatosis cases. Quant Imaging Med Surg. (2025) 15(4):3347–59. 10.21037/qims-24-1724 40235766 PMC11994522

[31] Kan H Cao Y Chen Y Zheng Y. Intravenous leiomyomatosis complicated by arteriovenous fistula: case series and literature review. Front Cardiovasc Med. (2022) 9:878386. 10.3389/fcvm.2022.878386 35770232 PMC9234661

[32] Baltodano-Arellano R Falcón-Quispe L Cupe-Chacalcaje K Meléndez-Ramírez G Cachicatari-Beltran A Patrón-Chi S. Collection of cardiac masses. Up-to-date echocardiography and cardiac MRI tools. Echocardiography. (2024) 41(2):e15757. 10.1111/echo.15757 38411212

[33] Castro-Martín JJ Di Silvestre-Alonso MA Rivero-García M Muñoz-Rodríguez R Izquierdo-Gómez MM Baeza-Garzón F. Magnetic resonance imaging in the study of cardiac masses: a case series. Medicina (B Aires). (2023) 59(4):705. 10.3390/medicina59040705 PMC10144986 37109663

[34] Bauer WR. Cardiac MRI today. Dtsch Med Wochenschr (1946). (2021) 146(5):344–50. 10.1055/a-1239-5068 33648004

[35] Sarra BL Ghalleb M Bouida MA Rachdi MM Ayedi MA Dhieb TB. Case report: benign yet aggressive: intravascular extension of leiomyoma. Int J Surg Case Rep. (2025) 134:111745. 10.1016/j.ijscr.2025.111745 40795718 PMC12361626

[36] Wang Y Cui Y Chen Y Lan H. Differential diagnosis of thrombosis and myxoma in unusual heart positions by dual-energy CT multiple parameter imaging: a case series. Medicine (Baltimore). (2025) 104(4):e41303. 10.1097/MD.0000000000041303 39854739 PMC11771713

[37] Kir G Cetiner H Gurbuz A Eren S. Immunohistochemical profile of intravenous leiomyomatosis. Eur J Gynaecol Oncol. (2004) 25(4):481–3. 15285309

[38] Hwang Y Cha SH Kim D Jun H-S. Combination of PD98059 and TGF-β1 efficiently differentiates human urine-derived stem cells into smooth muscle cells. Int J Mol Sci. (2021) 22(19):10532. 10.3390/ijms221910532 34638875 PMC8508912

[39] Smyth LCD Rustenhoven J Scotter EL Schweder P Faull RLM Park TIH. Markers for human brain pericytes and smooth muscle cells. J Chem Neuroanat. (2018) 92:48–60. 10.1016/j.jchemneu.2018.06.001 29885791

[40] McCluggage WG Sumathi VP Maxwell P. CD10 Is a sensitive and diagnostically useful immunohistochemical marker of normal endometrial stroma and of endometrial stromal neoplasms. Histopathology. (2001) 39(3):273–8. 10.1046/j.1365-2559.2001.01215.x 11532038

[41] Tsuchihashi H Ito A Tsukada H Hasegawa H Naitoh H Hanasawa K. A case of amelanotic anorectal malignant melanoma mimicking gastrointestinal stromal tumor. Gan To Kagaku Ryoho. (2011) 38(13):2659–62. 22189238

[42] Hua J Auw-Hädrich C. Combined squamous cell carcinoma and malignant melanoma of the eyelid. Klin Monbl Augenheilkd. (2008) 225(8):723–6. 10.1055/s-2008-1027180 18712658

[43] Danen EHJ van Rheenen J Franken W Huveneers S Sonneveld P Jalink K. Integrins control motile strategy through a Rho–cofilin pathway. J Cell Biol. (2005) 169(3):515–26. 10.1083/jcb.200412081 15866889 PMC2171933

[44] Lim MJ Whitney JE Sallee CJ Markovic D Bera A Sinha P. Plasma soluble intercellular adhesion molecule-1 has a central role in biomarker network analysis and is associated with poor outcomes in two distinct pediatric cohorts of acute respiratory distress syndrome and acute respiratory failure. Crit Care Med. (2025) 53:e1457–69. 10.1097/CCM.0000000000006719 40459371 PMC12323721

[45] Howell J A Candelario-Jalil E. "Gelatin zymography to quantify levels of MMP-2 and MMP-9 in Complex biological samples". Zymography. Biological and Clinical Applications, Volume 2. New York, NY: Springer US, (2025). 47–59. 10.1007/978-1-0716-4482-9_5 40261613

[46] Song J Lee Y Kim MS Ha G Jang W Batjargal U. High throughput drug screening platform utilizing capillary and artery cell layered models based on tumor–vascular cell interactions. Lab Chip. (2025) 25(10):2349–63. 10.1039/D4LC00950A 40177711

[47] Orimo A Gupta PB Sgroi DC Arenzana-Seisdedos F Delaunay T Naeem R. Stromal fibroblasts present in invasive human breast carcinomas promote tumor growth and angiogenesis through elevated SDF-1/CXCL12 secretion. Cell. (2005) 121(3):335–48. 10.1016/j.cell.2005.02.034 15882617

[48] Fukumura DAI Duda DG Munn LL Jain RK. Tumor microvasculature and microenvironment: novel insights through intravital imaging in pre-clinical models. Microcirculation. (2010) 17(3):206–25. 10.1111/j.1549-8719.2010.00029.x 20374484 PMC2859831

[49] Zhang Y Wu X. Treatment of intravascular leiomyomatosis: case report and literature review. J Vasc Surg Cases Innov Tech. (2023) 9(2):101059. 10.1016/j.jvscit.2022.10.017 37152917 PMC10160508

[50] Masood I Duran C Malik K Frank L. Uterine intravenous leiomyomatosis with cardiac involvement. Radiol Case Rep. (2020) 15(8):1389–93. 10.1016/j.radcr.2020.05.053 32636980 PMC7329926

[51] Sato S Yamanaka K Hashimura Y Ichikawa M Tara Y Nakatsuka D. Surgical strategy of intravenous leiomyomatosis with intracardiac extension: a case report. Ann Vasc Dis. (2025) 18(1):cr.25-00062. 10.3400/avd.cr.25-00062 PMC12406148 40909765

[52] Xie T Masroor M Liu C Lin S Song J Wang Z. Resection of intracardiac leiomyoma originating from the inferior vena cava through a single median sternotomy incision using a silk suture snare technique: a case report. BMC Cardiovasc Disord. (2023) 23(1):592. 10.1186/s12872-023-03630-z 38036979 PMC10691141

[53] Tânia B Catarina MS Sofia MO Mariana PP António RV Carolina PL. Intravenous leiomyomatosis: the importance of an early diagnosis. Cureus. (2025) 17(5):e84486. 40539160 10.7759/cureus.84486 PMC12178541

[54] Masarova L Novak J Pesl M Ondrasek J Semenka J Simarova E. Reccurent thrombus in the gigantic left atrium during effective anticoagulant therapy: case report. BMC Cardiovasc Disord. (2020) 20(1):86. 10.1186/s12872-019-01279-1 32085730 PMC7035782

[55] Traub A Vorwerk J Morf J Baumgarten N Agaimy A Horn M. Intravascular lipoleiomyoma with expansion into the right atrium and subsequent debulking surgery. Case Rep. (2025) 30(15):103764. 10.1016/j.jaccas.2025.103764 PMC12198623 40541356

[56] Mathey MP Duc C Huber D. Intravenous leiomyomatosis: case series and review of the literature. Int J Surg Case Rep. (2021) 85:106257. 10.1016/j.ijscr.2021.106257 34343794 PMC8350006

